# Genome-Wide Identification and Analysis of *DOF* Gene Family in *Eugenia uniflora* L. (Myrtaceae)

**DOI:** 10.3390/genes13122235

**Published:** 2022-11-28

**Authors:** Edgar Luis Waschburger, Frank Guzman, Andreia Carina Turchetto-Zolet

**Affiliations:** 1 Programa de Pós-Graduação em Genética e Biologia Molecular (PPGBM), Departamento de Genética, Instituto de Biociências, UFRGS, Av. Bento Gonçalvez 9500, Porto Alegre 91509-900, Brazil; 2 Escuela de Medicina, Universidad Científica del Sur, Lima 15067, Peru

**Keywords:** *DOF*, transcription factor, phylogeny, molecular evolution, native species, neotropics, adaptation, diversity

## Abstract

*Eugenia uniflora* is a Brazilian native plant species with great ecological and economic importance. It is distributed throughout the Atlantic forest, where two distinct populations show local adaptation to the contrasting conditions of restinga and riparian forest. Among various TFs described in plants, the *DOF* TF family has been reported to affect flowering and vascular development, making them promising candidates for characterization in *E. uniflora*. In this study, 28 *DOF* genes were identified by a genome-wide analysis, of which 20 were grouped into 11 MCOGs by Bayesian phylogeny, suggesting a shared functionallity between members. Based on RNA-seq experiments, we have detected eight drought responsive genes, and SNPs identification revealed population unique polymorphisms, implying a role in local adapatation mechanisms. Finally, analysis of conserved motifs through MEME revealed 15 different protein motifs, and a promoter region analysis returned 40 enriched TF binding motifs, both reporting novel biological functions circa the *DOF* gene family. In general, the *DOF* family is found to be conserved both in sequence and expression. Furthermore, this study contributes to both *DOF* literature and the genetic exploration of native species, elucidating their genetic potential and bringing to light new research topics, paving the way to future studies.

## 1. Introduction

**Figure 1:**
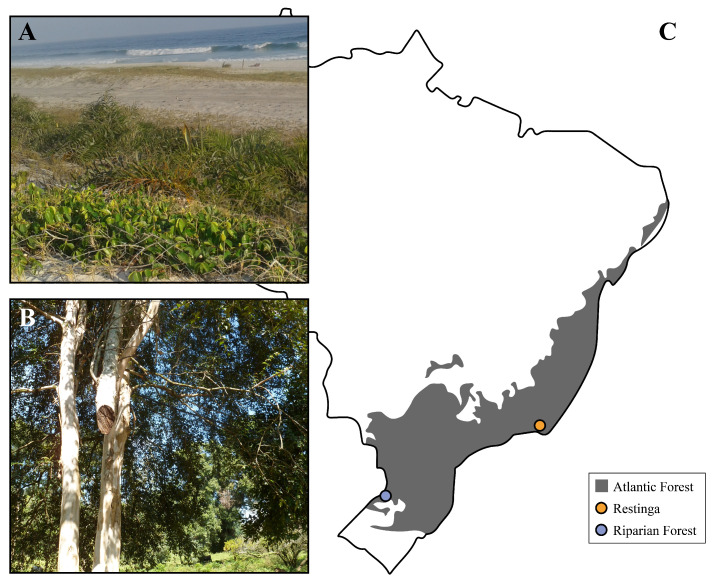
*Eugenia uniflora* individuals along the Atlantic Forest. (**A**) Restinga. (**B**) Riparian Forest. (**C**) Atlantic Forest dispersion along the Brazilian coast. Sampling points are depicted by colored dots.

The Atlantic Forest (AF) is a South American biome estimated to host over 15,000 different plant species [1], most of them belonging to the Myrtaceae botanical family [2], which includes the *Eugenia*, *Psidium*, *Syzigium* and *Eucalyptus* genera. One of its representatives, *Eugenia uniflora* L., also known as “Brazilian cherry tree”, is a model plant for genetic and biodiversity studies [3,4]. It is a small- to medium-sized woody tree reaching up to 12 m under optimal environmental conditions. Blooming mainly in spring, it gives rise to small, white flowers, which can undergo cross-pollination or self-fertilization, yielding ripe fruits of yellow, red and black colors. The leaves and fruits of *E. uniflora* are rich in vitamin A, B and C, lycopene, calcium, iron, phosphorus, anthocyanins and flavonols. The extracts of these tissues are widely used in folk medicine and have many biological activities described in the literature [5,6,7,8,9]. *Eugenia uniflora* presents local adaptation to different ecoregions with contrasting characteristics within the AF (Figure 1C) [10]. It presents itself as a shrub in the Brazilian Restinga (RE), coastal location of sandy and saline terrain, strong insolation and intense gusts of wind (Figure 1A), while growing to be 5 m tall in the Riparian Forest (RF), characterized by closed, humid and tropical vegetation (Figure 1B). This disparity between populations was also confirmed at the genetic level, where individuals from the RE had much smaller genetic variation compared to RF individuals, possibly due to a recent population expansion of individuals adapted to the conditions of the RE [11].

*DNA-binding with one finger* (*DOF*) proteins are part of a transcription factor (TF) family particular to the Viridiplantae clade [12]. They have been identified in numerous species, including monocots (rice, maize and sorghum) [13,14,15], eudicots (arabidopsis, cotton and tomato) [16,17,18], ferns (*Selaginella moellendorffi*) [12], mosses, (*Physcomitrella patens*) [19], and algae (*Chlorella vulgaris* and *Chlamydomonas reinhardtii* [12,20]). These proteins are mainly expressed in vascular tissues, and *DOF* over-expression studies have reported higher fruit yields, making them compelling candidates for characterization studies [21]. *DOF* TFs have a highly variable number of representatives, ranging from 1 in *C. reinhardtii* to more than 100 in polyploid species, such as cotton [12,17]. This diversity is the result of multiple duplication events at the genetic and genomic levels [17]. Sequences range from 200 to 500 amino-acids and are characterized by a C2C2 zinc-finger DNA-binding domain [22], that specifically binds to 5’-(A/T)AAAG-3’ motifs [23] at the gene promoter region, and at least one other domain for protein interaction. They have already been described as regulators of several physiological functions, such as flowering [24], lipid metabolism [20], starch synthesis [25], regulation of development in response to abiotic stresses [26,27], acclimation to cold [28] and others [29,30,31].

As aforementioned, *E. uniflora* individuals exhibit contrasting phenological characteristics among their populations (RE and RF), some of which are maintained even when greenhouse-grown (e.g., different flowering periods). Thus, the characterization of *DOF* TFs (described as actuators in flowering and abiotic stresses), and their comparison of different populations is important both from an ecological and economical points of view (conservation, gene flow between populations and, since this species is cultivated, a phenological standardization is essential). The objective of the present study was to carry out a genomic characterization of the *DOF* gene family in *E. uniflora*, and as result, we have identified 28 *DOF* members in *E. uniflora*’s genome, further classified into 11 Majorly Conserved Orthologous Groups (MCOGs) by a phylogenetic approach. These MCOGs returned new phylogenetic groups not yet described, and brought to light the confounding *DOF* classification system used in the current literature. As for *E. uniflora* populations, *DOF* genes appear to be conserved both in sequence and expression profiles, serving as actuators in local adaptation.

## 2. Materials and Methods

### 2.1. Identification of *DOF* Genes in Eugenia uniflora

To identify *DOF* genes in *E. uniflora*, all *DOF* gene sequences from its closest phylogenetic species—*Eucalyptus grandis*—were used as query for BLASTp [32] search against the contigs of the *E. uniflora* genome (SUB10728242). Hits obtained, with an e-value below 1.00${}^{-5}$, were validated by manual filtering using transcript visualization and ORF prediction with Unipro UGENE [33], *ab initio* gene prediction with FGENESH [34], and visualization of hits of RNA-seq experiment alignment files with TABLET [35]. All sequences obtained were compared with the databases available at NCBI (ncbi.nlm.nih.gov, accessed on 29 October 2022) and Phytozome (phytozome-next.jgi.doe.gov, accessed on 29 October 2022) by BLASTp to confirm their existence.

### 2.2. In Silico Predictions

The Expasy server’s ProtParam online tool (web.expasy.org/protparam, accessed on 29 October 2022) was used to compute the chemical characteristics of *E. uniflora*. The subcellular location prediction was performed on the DeepLoc1.0 server [36]. The gene structure was manually assembled in Adobe Illustrator CC (adobe.com/products/illustrator.html, accessed on 29 October 2022). The MEME tool from the MEME-suite [37] was used to identify conserved protein motifs, 15 motifs between 6 and 100 amino-acids with one or no occurrence were predicted from all sequences present in the phylogenetic analysis.

### 2.3. Alignment and Phylogeny Reconstruction

Sequences of *DOF* genes were gathered from 16 species that have already been phylogenetically characterized. Included species in this study are: *Arabidopsis thaliana* [16], *Cajanus cajan* [38], *Camellia sinensis* [39], *Citrullus lanatus* [40], *Cucumis sativus* [41], *Daucus carota* [42], *Eucalyptus grandis* [43], *Manihot esculenta* [44], *Medicago truncatula* [45], *Musa acuminata* [46], *Oryza sativa* [13], *Populus trichocarpa* [47], *Selaginella moellendorffii* [12], *Solanum lycopersicum* [18], *Vitis vinifera* [48] and *Zea mays* [14]. The alignment used in the phylogeny used the protein sequences of the *DOF* genes and was generated by the MAFFT [49] tool. Not all sequences were kept for future analysis (Supplementary File S1). MEGAX [50] was used to visualize and remove sequences without the presence of the *DOF* domain. Only regions referring to the *DOF* domain and adjacent regions with a possible phylogenetic signal were considered for alignment. Columns with high representation of gaps were excluded from the final alignment. Gaps were converted to missing data. The most suitable evolutionary model for the resulting alignment was predicted by ModelFinder, included in the IQTree [51] package. The phylogenetic tree was constructed by Bayesian analysis in BEAST [52] using 50,000,000 generations, while discarding 10% of the most discrepant trees. The FigTree program (tree.bio.ed.ac.uk/software/figtree, accessed on 29 October 2022) was used to visualize and manipulate the trees.

### 2.4. Differential Expression Analysis, SNP Indentification and Promoter Motif Enrichment Analysis

**Figure 6:**
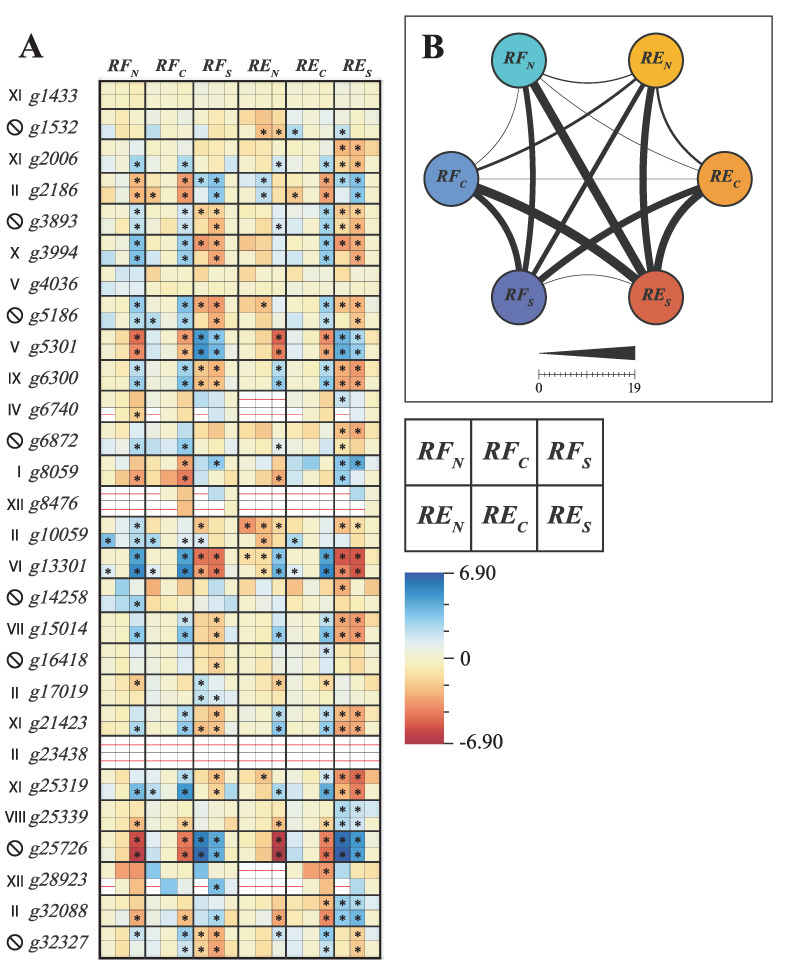
Differential gene expression analysis of *Eugenia uniflora DOF* genes. (**A**) Each gene phylogenetic groups is indicated on its left. Ungrouped genes are indicated by a crossed-out “O”. Asterisks represent statistically significant FCs, while red lines represent no expression. Each column refers to a sample condition: Riparian Forest in natura (RF${}_{N}$), RF Control (RF${}_{C}$), RF Stressed (RF${}_{S}$), Restinga (RE)${}_{N}$, RE${}_{C}$, and RE${}_{S}$. Each gene has six comparisons on every column, exemplified by the scheme over the FC color gradient. (**B**) DEGs comparison amongst every condition. Thicker lines represent more DEGs.

**Figure 7:**
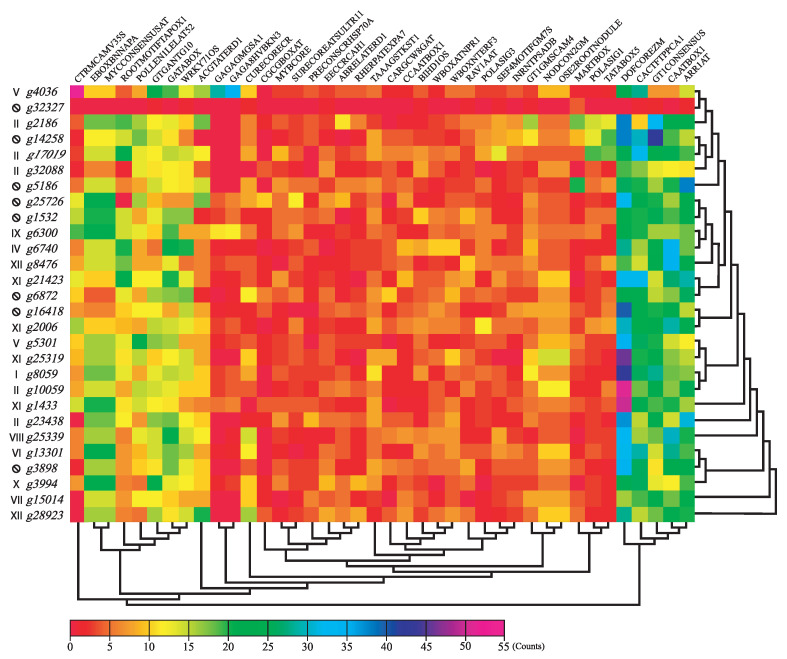
Motif enrichment analysis of the promoter region of *Eugenia uniflora DOF* genes. Rows represent *DOF* genes while columns are motifs found. Each gene has its phylogenetic group represented on its left. Crossed-out “O”s represent ungrouped genes. Both genes and motifs were clustered using their count profiles, denoted by the cladograms on the bottom and right.

The raw transcriptome datasets of *E. uniflora* leaves were recovered from the NCBI Sequence Read Archive at Bioproject PRJNA784246 (Turchetto-Zolet et al. unpublished). Drought experiment reads of plants in natura, controlled conditions, and under drought treatment were aligned against *E. uniflora* genome contigs using the STAR software package [53]. SNP identification was performed using Picard (broadinstitute.github.io/picard, accessed on 29 October 2022) followed by the GATK best practice workflow for variant identification (gatk.broadinstitute.org/hc/en-us, accessed on 29 October 2022). Differential expression analysis was performed using DESeq2 package [54] and promoter region analysis was performed on the NewPlace database [55]. Heatmap plots were assembled from Fragments per kilobase per million (FPKM) values—differential expression analysis (Figure 6)—and raw number of motifs found in the 2 kpb upstream region of *DOF* genes starting from the coding sequence (CDS)—promoter motif analysis (Figure 7). Differentially expressed genes were selected according to their fold-change (FC) values > 2. Only motifs that presented more than 8 copies were included in the promoter analysis heatmap. Both axes were grouped by the minimum distances of their profiles using the UPGMA algorithm. The Python data visualization toolkit Matplotlib [56] and Adobe Illustrator CC illustration were used to generate the figures.

## 3. Results

### 3.1. Identification and Classification of *E. uniflora* DOF Genes

A total of 32 genes were identified in *E. uniflora*, of which 28 remained after manual filtering steps (Table 1). Proteins cover sizes between 186 and 507 residues (g8059 and 2186), with most proteins being between 250 and 350 residues. The majority of proteins had their isoelectric point (pI) above 7.0, with the exception of 8 proteins (g1532, g2186, g3994, g5186, g8476, g15014, g23438, g28923). All proteins were predicted to be located in the cell nucleus, as to be expected from typical transcription factors.

**Figure 2:**
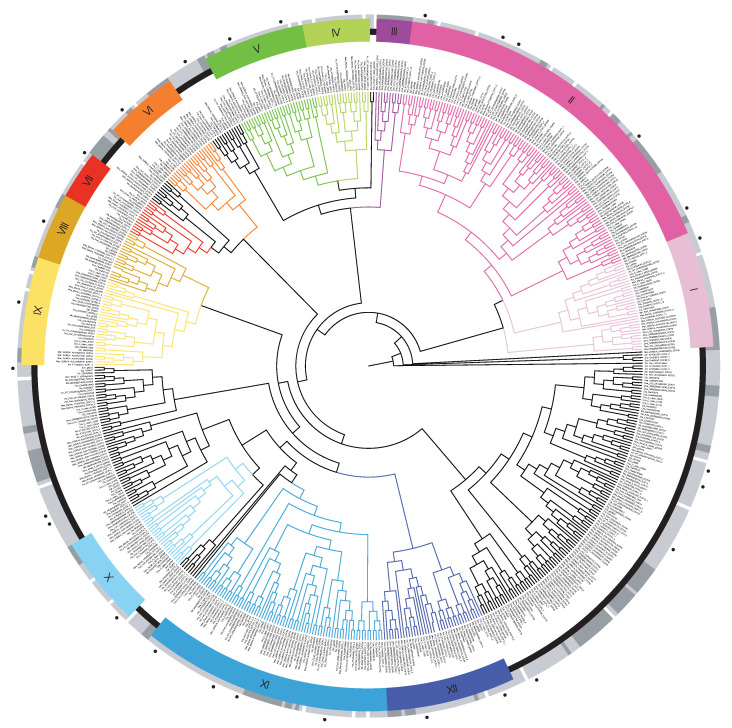
Phylogenetic tree of 615 *DOF* amino-acid sequences. Conserved clades with species representativity are colored, together with their branches. Outer circle colors denote sequences derived from monocots (dark grey), eudicots (light grey), and *Selaginella moellendorffii* (white). Black dots indicate *Eugenia uniflora* sequences.

A total of 647 sequences covering the 16 species studied were recovered from the different databases used in each study, of which 32 sequences were removed for various reasons (Supplementary File S1)—including the lack of the *DOF* domain—leaving 615 sequences for further analyses. The total size of the alignment was manually curated to include only 61 sites, covering the *DOF* domain and adjacent regions with a possible phylogenetic signal. The tree generated by Bayesian statistics (Figure 2) returned 12 different MCOGs with high support—Posterior Probability (PP) > 0.94—and representativeness of sequences from different species.

Among the recovered groups, the largest was subdivided into two groups (Group I, representing the Cycling *DOF* Factors (CDF) subfamily of *DOF* genes, and II). Group II, even though paraphyletic, was considered to be just one group. The reasoning was based on species representativity (each of its divisions represents only monocot or eudicot species) and a PP equal to 1 on the branch that refers to Groups I and II. Groups VI and XII also presented PP equal to 1. Group III is another group with only monocot species. In general, it was not possible to determine the evolutionary relationships between MCOGs, given by the low support values between the groups, with the exception of Groups I and II. Of the 28 genes of *E. uniflora*, 20 were grouped into 11 MCOGs (Groups I–II and IV–XII), with 5 sequences present in the CDF group, while 8 were not grouped (*g25726, g6872, g5186, g1532, g14258, g32327, g3898* and *g16418*). Among the sequences present in the conserved groups, *g2186, g6740, g15014, g6300, g25319* and *g8476* did not have their closest ortholog being an *Eucalyptus grandis* gene. Regarding the ungrouped sequences, *g32327* and *g16418* also did not cluster with *Eucalyptus grandis*.

### 3.2. Gene Structure and Domain Conservation of E. uniflora DOF Genes

Among the 28 genes identified in *E. uniflora*, none had more than 1 intron in their gene structure, while 13 had no introns at all (*g1433, g4036, g5301, g6740, g8059, g8476, g13301, g14258, g15014, g25339, g25726, g28923, g32327*) (Supplementary Figure S1). The CDF subfamily returned the largest intron sizes, with a mean of nearly 1000 nucleotides. Some sequences had exons with only a few dozen nucleotides, mainly in the N-terminal region, with the exception of the g1532 sequence, which had a small exon in its C-terminal region. In general, the different MCOGs found seem to be represented in the genetic structure of their representative sequences.

**Figure 3:**
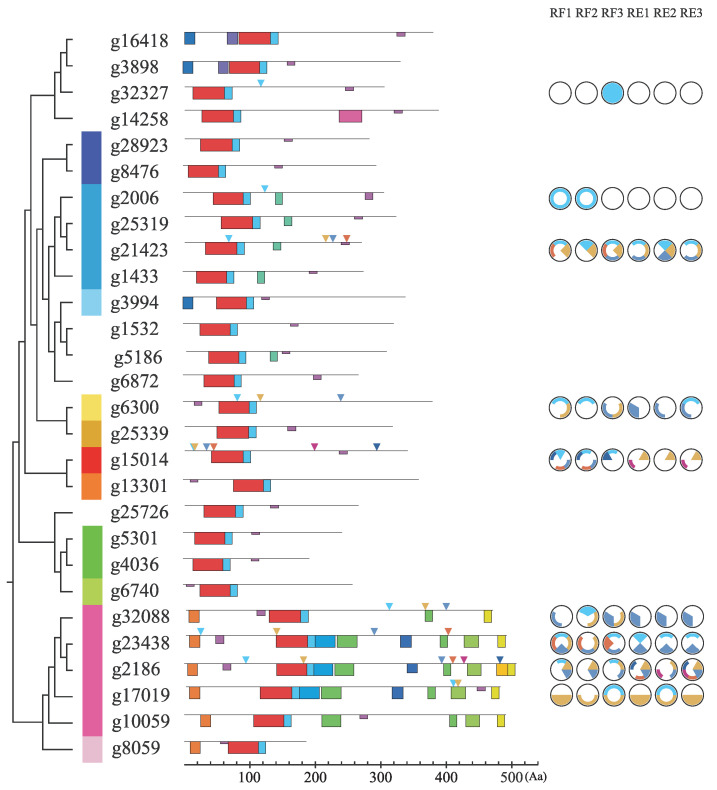
Conserved protein motifs and SNPs from *Eugenia uniflora DOF* genes. The phylogenetic relationships between *DOF* genes are shown on the left. Colored boxes represent protein motifs. Black lines represent protein sequences without annotated motifs. Triangles indicate identified SNPs locations. On the right, a comparison of SNPs between the two populations is shown. Filled slices represent homozigote polymorphisms and outlined slices heterozigotes ones.

**Figure 4:**
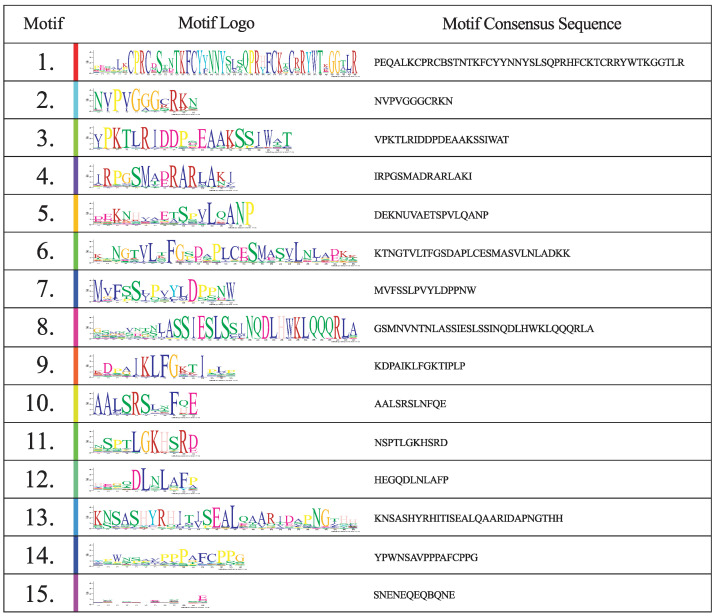
Conserved protein motifs identified in *Eugenia uniflora DOF* genes. Motifs are ordered based on false discovery rate, from lowest to highest.

Of the 15 protein motifs identified in the protein sequences of *DOF* genes (Figure 3 and Figure 4), sizes vary, from small motifs of 11 amino-acids (motifs 2, 9, 10 and 11) up to motifs with 46 amino-acids (motif 1).

The vast majority of proteins had 3 or 4 motifs found in their sequence, with the exception of CDF proteins (g2186, g10059, g17019, g23438, g32088), which had up to 11 motifs found. Motifs 3, 9, 10 and 11 are typical motifs present in CDF proteins and have already been characterized as responsible for protein-protein interaction with other participants in the flowering regulation process, such as FLAVIN-BINDING KELCH REPEAT F BOX PROTEIN (FKF1), TOPLESS (TPL) and GIGANTEA (GI). Motifs 5, 6, 13 and 14 were also found exclusively in the CDF proteins and in more than one representative, although their functions are unknown. Motif 12 appears to be conserved in sequences belonging to Group XI, also with unknown function. Apart from motif 15, all the other motifs showed considerable conservation of their residues.

### 3.3. E. uniflora Populations and Their DOF Gene Arsenals

**Figure 5:**
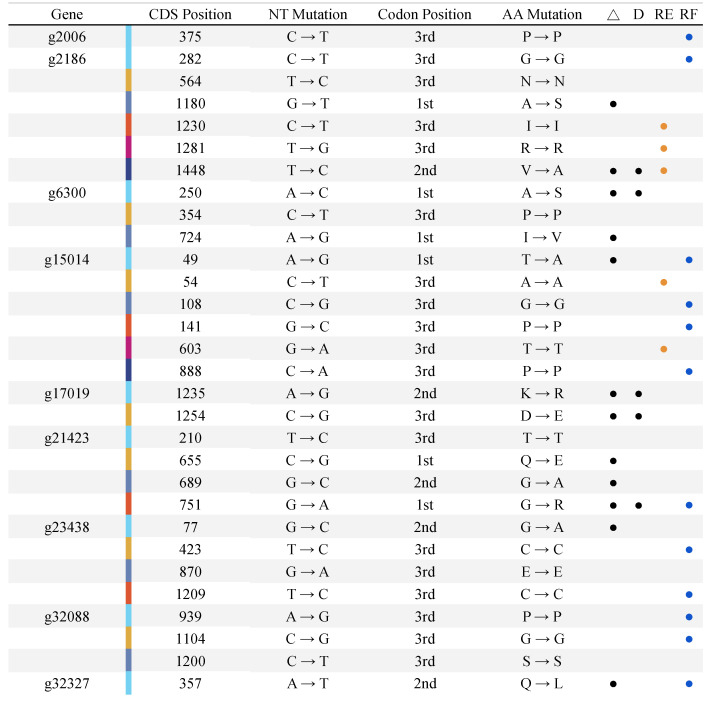
Characteristics of the 30 identified *DOF* SNPs in *Eugenia uniflora*. For every SNP, the gene, CDS mutation position, nucleotide mutation, codon position, and amino-acid changes are shown. The last four columns indicate, in order, a non-synonymous mutation (Δ), a mutation inside a motif (D), RE specificity, and RF specificity.

A total of 30 high quality SNPs were identified from expression data of the two populations (Figure 3 and Figure 5), but with only 9 SNPs resulting in non-synonymous mutations. Of the 28 genes, two (*g2186* and *g15014*) had 6 SNPs each, the highest number identified among all sequences. SNPs were identified in four of the five genes representing Group I. Seventeen SNPs are specific to only one of the populations (12 from the RF population and 5 from the RE population). Almost two thirds (18) of the identified SNPs are found in the third position of the codons referring to the reading phase of the *DOF* genes, all being synonymous mutations, except for the second SNP of the *g17019* gene. Of the remaining 13 SNPs, 5 are located in the regions of the motifs found (g21423;4, g6300;1, g2186;6, g17019;1/2). Three SNPs are exclusive to RF individuals (g32327, g21423;4, g15014;1) and 1 to RE individuals (g2186;6).

Regarding the differential expression experiment, of the 28 genes, 4 (*g6740, g8476, g23438* and *g28923*) were not expressed in at least one analyzed condition studied, and 4 genes (*g1433, g4036, g8476* and *g23438*) did not have their expression altered between the two populations or between treatments (Figure 6A). Of the 24 differentially expressed genes (DEGs) in at least one of the conditions, 8 have a clear drought responsive profile, 5 of which are upregulated (*g3893, g3994, g5186, g6300, g13301*) and 3 are downregulated (*g2186, g5301, g25726*). Genes *g5301, g13301* and *g25726* are the three most differentially expressed, with FC values around 4.9, 4.7 and 6.9 respectively. Overall, control conditions (RF${}_{C}$ and RE${}_{C}$) and the in natura RF (RF${}_{N}$) have little variance amongst each other, also true for both RF and RE drought stress conditions (RF${}_{S}$ and RE${}_{S}$) (Figure 6B). The highest number of DEGs was found when comparing RE${}_{S}$ with RF${}_{N}$ (19).

### 3.4. Promoter Region Analysis

All *Eugenia uniflora DOF* genes had their upstream 2 kbp, starting from the CDS region, used for promoter analysis with the exception of two genes: *g32088* and *32327*, which were recovered only 1327 and 138 bp respectively due to their close location to the 5’ region of the genome contigs.

A total of 290 unique motifs were identified, of which 40 were left after filtration steps (Figure 7). The number of copies per motif varies from 0 to 55 copies, the case of the CTRMCAMV35S motif. Among the motifs found in the promoter region of *DOF* genes, sequences related elements of the transcription machinery, such as TATABox and CAATBox, were enriched. Recognition sequences of different transcription factors were also well represented, some already mentioned in both *in silico* and *in vivo* studies of the promoter region (WRKY, ABRE, MYB, BELL) [30,41,47,57,58,59], with the vast majority yet to be confirmed (CAMTA3, BBR/BPC, SURE). Among the tissue-specific-expression elements found, primary and secondary root, vascular tissues, leaves, seeds, flowers and pollen are already mentioned in the literature [60], however we also identified a nodule specific motif. As for the biological function, three different motifs for light induction were found (PRECONSCRHSP70A, INRNTPSADB and GT1CONSENSUS). Other motifs include responses to sulfur, calcium, copper, chlorophyll precursors, etiolation, drought, salinity, pathogen induction, and vernalization. Interestingly, the motif that was the most represented over all *DOF* genes was not related to classical transcriptional machinery, such as CAATBox, CCAATBox or TATABox, but the *DOF* recognition motif itself.

## 4. Discussion

### 4.1. Phylogenetic Relationships Reveal DOF Genes Acting in Similar Pathways

Twenty eight *DOF* genes were identified in *E. uniflora* with their physicochemical characteristics shown in Table 1. Their genetic structure was reconstructed and, overall, sequences shared a similar structure among orthologous groups identified in the phylogeny (Supplementary Figure S1 and Figure 2). Motifs 1 and 2, referring to the *DOF* domain, are present in all sequences; why they appear separately is not well understood, but it is a recurrent phenomenon in the prediction of conserved *DOF* sequence motifs through MEME [61,62]. From the reconstruction of phylogenetic relationships, eight *E. uniflora DOF* genes were not grouped into orthologous groups. However, the other 20 genes are present in clades with high support (PP > 0.94) and their functions could be inferred (Figure 2). The composition of the other groups vary greatly in the literature. Some are preserved, such as the case of Group VI [16,63], and the vast majority end up being subdivisions of larger groups, such as Groups X, XI (the second largest group) and XII [12,63]. These inconsistencies arise due to many methodological choices (e.g., the inclusion of only a few species, utilization of less refined phylogenetic methods neighbor-joining, and labelling of poorly supported groups, PP < 80), stating a need for more evolutionary focused studies circa the *DOF* TF family.

Five genes (*g2186, g10059, g17019, g23438* and *g32088*) were grouped together with Group II, referring to the CDF subfamily of *DOF* genes, which represents the average number of CDF genes found by species, with some exceptions [64]. CDFs are *DOF* genes that act in the repression of *CONSTANS* (*CO*) and *FLOWERING LOCUS T* (*FT*) and thus in flowering inhibition. They have also been characterized to respond to abiotic stresses [65]. Their protein interactions and regulation networks, such as ubiquitination by the GI/FKF1 complex, are well explored in the literature [66]. CDF grouped genes of *E. uniflora* also contain domains 3, 9, 10 and 11, for interaction with FKF1, TPL and GI respectively, with only the function of motif 11 yet to be discovered [64,67,68,69], corroborating with their possible role as flowering regulators (Figure 3 and Figure 4). Interestingly, 3 other motifs with no described function (5, 6 and 13) also appear conserved in *E. uniflora* CDF genes. The same three motifs are organized in such a way that there are 4 represented combinations among the 5 CDF genes, (No domains, 6, 6 + 13, and 5 + 6 + 13). This may be a diversification of CDF genes to interact with other proteins and act in other signaling pathways that are not flowering, as all have been described as pleiotropic genes in CO and FT inhibition [24]. Group I holds genes orthologous to CDF genes, but with very different functionalities. In past phylogenetic studies, Groups I and II were considered as a single group [13,16], but recently, phylogenies with higher sequence numbers present these groups separately, even if in lower support clades [19,63]. In this study, Group I was found with a PP equal to 1, indicating that these genes, despite having a probable common ancestor with the CDF genes, are not part of the same functional group. The *g8059* gene was grouped into Group I, together with sequences from A. thaliana, such as *AtDOF1.5* (*AT1G29160/COG1*) and *AtDOF2.3* (*AT2G34140/CDF4*), the only ones experimentally characterized between eudicots and monocots. COG1 and CDF4 are transcription factors involved in the inhibition of phytochrome response pathways, both phytochrome A and B (phyA and pyhB), and the transduction of these signals to hormonal pathways, such as the synthesis of gibberellic acid (GA), abscisic acid (ABA) and brassionosteroids (BRs) [70,71,72,73]. Motif 9, referring to the TPL corepressor protein binding domain, was also identified as conserved in Group I sequences, probably acting as a key domain for the action of its repressor activities. The presence of this domain in both Group I and Group II genes is further evidence of their likely common ancestry. The most likely hypothesis is the formation of Group I from gene duplications in the ancestors of these sequences and subsequent loss of its C-terminal region by negative or neutral selection, thus maintaining the TPL-binding domain. As with *Eucalyptus grandis*, *M. truncatula* and *S. moellendorffii*, only one sequence was identified in Group I, indicating a convoluted evolutionary history, since other species usually present two, possibly marked by pseudogenization and gene duplication events.

Group III, also reconstituted by other phylogenies (d3) [13], presents only monocot sequences, which may represent an evolutionary novelty exclusive to this clade. Although one *M. acuminata* sequence is present, this clade might be an exclusivity to Poales, after all, the genes of *M. acuminata* tend to be grouped in a greater number of sequences. A phylogeny with a greater number of species representing monocots would be ideal for a proper investigation. Only the rice *OsDOF18* gene (*LOC_Os08g38220*) was characterized in this group, acting on the inhibition of genes of the jasmonic acid (JA) biosynthesis pathway and chlorophyll degradation in leaves and on inducing the expression of ammonia transporters in roots [31,74]. Although they seem to be two uncorrelated pathways, studies evaluating soil nitrogen availability and JA biosynthesis found changes in the expression of the same genes that *OsDOF18* regulates [75]. On the other hand, Group IV did not present any sequence of monocots, only eudicots and the lycophyte S. moellendorffii, indicating a possible loss of this MCOG in cereals. The gene *g6740* was grouped with the sequence *DOF5.8* (*AT5G66940*), characterized by the expression in procambium and provascular cells, related to the presence of auxin in the plant, since *DOF5.8* is regulated by *MONOPTEROS* [76,77]. *DOF5.8* also regulates the expression of *ANAC069,* an auxin-responsive factor responsible for controlling responses to abiotic stresses such as salinity [78]. Group V has two *E. uniflora*, *g4036* and *g5031*, grouped with two other *A. thaliana* genes, *AtDOF1.7* (*AT1G51700/ADOF1*) and *AtDOF3.1* (*AT3G21270/ADOF2*). Although there is not much information about the function of these two genes in specific characterization studies, multi omics-data have returned both genes as differentially overexpressed in the overexpression of factors involved in cell division and vascular tissue development (TDIF-PXY) [79]. The same study also detected genes regulated by *ADOF1* (*LBD4, PHB, PHV* and *REV*) and by *ADOF2* (*REV*) from a Y1H analysis. Apart from the *LBD4* gene, the genes regulated by ADOFs belong to the HD Zip III family, responsible for mediating auxin responses, formation of leaf axes and maintenance of the shoot apical meristem. Furthermore, *ADOF1* was found to be overexpressed in roots of plant subjected to salinity stress [80]. Peculiarly, another study identified *ADOF1* mRNA to be expressed mainly in the aerial tissue, later mobilized to the root system [81].

Group VI has one *E. uniflora* gene (*g13301*) and no *A. thaliana* sequences, the same case for Group VIII with the *g25339* gene. The only characterized gene in Group VI is *OsDOF28* (*LOC_Os03g55610*), responsible for root apical meristem cell division by inhibition of *OsACS1*, responsible for ethylene biosynthesis [26]. In contrast to the promotion of cell division in the root of Group VI, Group VII groups genes *g15014* and *AtDOF5.4* (*AT5G60850*), the later functionally characterized as an inhibitor of cell cycle progression in callous cells by repression of expansins, cyclins, kinase-dependent cyclins and xyloglucan endo-transglycosylation enzymes [82]. It has also been shown to be induced by ABA and responsible for inhibiting the growth of secondary roots by regulating *RSL280*. Group IX has genes *g6300* and *AtDOF1.4* (*AT1G28310*), however no sequences are characterized. Another gene related to vascular development is *AtDOF5.6* (*AT5G62940/HCA2*) present in Group X, together with the sequence *g3994*. The generation of dominant mutants for the *HCA2* led to malformation of the interfascicular cambium and periclinal divisions of its cells [83].

Group XI has five *A. thaliana* genes *AtDOF1.8* (*AT1G64620*), *AthDOF2.5* (*AT2G46590/DAG2*), *AthDOF3.7* (*AT3G61850/DAG1*), *AtDOF4.1* (*AT4G00940/ITD1*) and *AtDOF4.6* (*AT4G24060*) grouped with four genes from *E. uniflora* (*g1433, g2006, g21423* and *g25319*). *DAG1* and *DAG2* were characterized in mutants with altered germination times. Mutants for *DAG1* show a lower need for incidence of red light for germination, while mutants for *DAG2* demonstrate the opposite [84]. Phytochrome Interacting Factor3-Like 5 (PIL5), responsible for inhibiting the germination process, is degraded via phyB-mediated ubiquitination and forwarding to the 26S proteasome. PIL5 induces the expression of members of the DELLA family, such as *GA Insensitive* (*GAI*) and *Repressor of ga1-3* (*RGA*), inhibitors of the GA synthesis pathway and essential for the maintenance of endogenous levels of ABA [85,86]. The *DAG1* gene has been shown to be related to the expression of *PIL5*, suggesting an induction of *DAG1* by PIL5 [87]. It is known that the interaction between DAG1 and GAI is necessary for the repression of *AtGA3ox1*, a gene of the GA biosynthesis pathway [88]. DAG1 has also been shown to inhibit the expression of *CYP707A2*, the ABA catabolic pathway gene, demonstrating similarity with DELLA proteins functions [89]. On the other hand, *DAG2* is inhibited by the expression of *PIL5* and *DAG1*. As for the genes regulated by DAG2, *RGA* appears to be negatively regulated, while the GA biosynthesis genes: *GA3ox1* and *GA3ox2* are shown to be induced, however it is not known whether this interaction is direct or not [90]. In general, it is proposed that the *DAG1* and *DAG2* genes act in the inhibition of germination by GA catalysis and inhibition of ABA catalysis. As for the *ITD1* gene, its function is not known, but its expression pattern shows localization in the cells of the cortex and endoderm of the root [91]. Furthermore, it has an intercellular traffic domain that overlaps with the *DOF* domain, allowing its dispersion from the expression region to nearby tissues by diffusion via plasmodesmata, a fundamental characteristic for the performance of some *DOF* genes involved in the development of vascular tissues [92]. All sequences present in Group XI showed domain 12. The *AtDOF1.8* and *AtDOF4.6* genes are not characterized. Group XII has two *A. thaliana* genes: *AtDOF1.2* (*AT1G21340*) and *AtDOF3.5* (*AT3G52440*) together with *E. uniflora* genes (*g8476* and *g28923*).

### 4.2. DOF Genes as Drivers for Local Adaptation

Monitoring greenhouse-grown *E. uniflora* plants, individuals coming from the RE showed a shorter flowering time when compared to individuals coming from the RF. In order to search for genetic variants linked to *E. uniflora* populations and that are also related to alterations in the functionality of *DOF* genes, 30 SNPs were identified, of which 2 population specific markers resulted in non-synonymous mutations (Figure 5). The g21423;4 SNP, exclusive to RF individuals, is located in motif 15, a highly variable region between the *DOF* sequences and most likely does not cause a functional change in the context of the *g21423* gene protein, belonging to Group XI of genes related to germination. In similar terms, the g2186;6 SNP, even being present in a CDF protein, occurs in a motif not yet characterized, and in a variable region within this motif. Both g17019 SNPs were obtained within the FKF1 binding domain and correspond to conserved amino-acid changes within the motif, however they are not unique to any population. The SNP g17019;1 was found only in heterozygosity, which may suggest a greater importance of conservation of the first lysine residue of this motif when compared to aspartic acid residues, but even if the binding function to FKF1 is compromised, the overlapping function of CDF genes would probably be enough to cover early flowering phenotypes. At first, the greenhouse observations cannot be explained by genetic variants present in *DOF* gene sequences. Perhaps a search for variants in regulatory regions such as the promoter will result in findings, but with a lack of motif characterization, further studies become difficult.

In order to understand the possible modulations of *DOF* genes, a gene expression analysis of plants under drought conditions was performed (Figure 6). *DOF* genes with the highest differential expression between control and drought-exposed conditions were genes *g5301*, *g13301* and *g25726*. Gene *g25726* was grouped with the *Egr.F00039* gene from *Eucalyptus grandis* (PP = 1), its closest ortholog and without known function. These genes may be a novelty exclusive to the Myrtaceae family, considering that both sequences were not present in conserved clades. The gene *g13301*, belonging to Group VI, was overexpressed under conditions exposed to drought. Ethylene, even being a phytohormone related to stress signals and orchestration of adaptive responses, is an auxin antagonist and its presence in the root stops the growth of its cells. In the context of physiological responses to drought, a stressful condition leads to a search for water by the roots. Inhibition of ethylene synthesis and promotion of apical root cell division in search for water sources may be one of the reasons for *g13301* differential expression, if its function is similar to its ortholog (*OsDOF28*). The third differentially expressed gene was *g5301*, representative of Group V. According to the previous gene, cell division of meristematic cells present in the root must be followed by mechanisms for differentiation and maturation, such as regulation of vascular development genes. Interestingly, its *g4036* ortholog was not differentially expressed, which may be due to a mechanism analogous to the specificity of the *AtDOF1.7* salinity response [80]. Although some sequences were not detected in leaf tissues, in general, CDF genes appear to be repressed in the drought treatment. Other members of conserved groups, such as *g25339* (Group VIII), *g15014* (Group VII), *g8059* (Group I), *g6300* (Group IX) and *g3994* (Group X) also showed changes in their expression by drought treatment. As for differences between populations in their natural environment, only genes *g10059* (Group II) and *g13301* (Group IV) showed differential expression. Possibly related to abiotic stresses of the RE environment, but with so many possibilities (salinity, drought, insolation) it is difficult to correlate with a specific response. Individuals from *E. uniflora* populations do not have alterations in the expression of *DOF* genes when greenhouse-grown, and based on their overall expression profiles, *DOF* genes seem to act as conserved tools to combat possible stresses present in the environment, rather than specialized pathways that confer an evolutionary advantage to individuals in a population.

The promoter region analysis (Figure 7) returned a total of 40 motifs described in the literature, with 9 repeats or more. The motif patterns found for each gene were grouped by clustering and the conserved orthologous groups were not recovered, indicating a high diversification of the promoter regions and their binding factors. The clustering of motif profiles returned two main groups: a group of 5 motifs with high representation in all *DOF* sequences (DOFCOREZM, CACTFPCCA1, GT1CONSENSUS, CAATBOX and ARR1AT), with an average of 20 or more repetitions, and another group representing the other 35 reasons and high variability. Among the most represented motifs, the presence of the DOFCOREZM and ARR1AT motifs indicate a high self-regulation of *DOF* genes, and a regulation by cytokinin-responsive factors, such as *ARR1*. The CACTFPCCA1 motif, specific to leaf mesophilic cells, and the GT1CONSENSUS motif, light signal transduction pathway regulation—such as *phyA*—indicate a wide expression of *DOF* genes in leaves. Finally, the CAATBOX motif is related to the recruitment of other transcription factors to the promoter region. Motifs for specific factors, such as *MYC* and *WRKY* genes, were highly represented, as were root and pollen tissue specific motifs. Some motifs have multiple copies in only a few genes, such as the *BBR/BPC* binding motif in the g4036 and g6300 gene, as well as the copper-response motifs present in the *g25339* and *g28923* genes.

## 5. Conclusions

In conclusion, 28 *DOF* genes were identified in *E. uniflora*, of which 20 were grouped into 11 phylogenetically conserved groups with a high probability for conservation of functionality. The eight remaining genes require a more robust phylogenetic analysis for proper grouping, yielding higher branch supports. The addition of more sequences would also be of interest for explaining MCOGs evolutionary relationships, since our results did not manage to do so. The analysis of conserved motifs corroborated with the data presented in the phylogeny, identifying new *DOF* protein motifs. It would be compelling to functionally characterize these same motifs and genes, possibly revealing new protein interactions and functionalities. With the differential expression analysis, we found eight drought-responsive *DOF* genes with little expression changes among populations. Hence appearing to be well-preserved tools that *E. uniflora* populations use to respond to environmental stresses. Future studies utilizing other tissues (e.g., roots and fruits) would also be of interest for providing a full picture of the local adaptation process. Finally, a comprehensive analysis of the promoter region revealed the diversity of regulatory regions of *E. uniflora DOF* genes, including previously unreported motifs, such as nodule and pollen specific which could be of interest for gene reported and promoter engineering studies. Overall, this work identified new *DOF* genes and brought to light aspects of their evolutionary history not yet addressed, presenting new avenues of research for the analysis of this family of genes in future analyzes.

## Figures and Tables

**Table 1:** Physicochemical properties and predicted subcellular location of *Eugenia uniflora DOF* proteins. Protein size is in amino-acid residues (Aa), molecular weight in kilo Daltons (kDa), and isoelectric point (pI) in pH.

Protein	Lenght (Aa)	Weight (kDa)	pI	Subcellular Location
g1433	276	29,822	8.85	Nucleus
g1532	331	36,445	6.52	Nucleus
g2006	307	33,047	8.79	Nucleus
g2186	507	55,049	6.10	Nucleus
g3898	332	35,571	9.19	Nucleus
g3994	340	36,687	6.72	Nucleus
g4036	193	21,308	7.55	Nucleus
g5186	306	32,971	6.74	Nucleus
g5301	243	26,111	8.54	Nucleus
g6300	381	41,256	8.70	Nucleus
g6740	259	26,047	8.45	Nucleus
g6872	268	28,331	9.38	Nucleus
g8059	186	20,727	9.62	Nucleus
g8476	295	31,875	5.87	Nucleus
g10059	491	52,622	8.37	Nucleus
g13301	360	38,370	9.07	Nucleus
g14258	370	38,939	8.82	Nucleus
g15014	325	34,705	6.51	Nucleus
g16418	362	38,399	9.59	Nucleus
g17019	458	49,638	6.87	Nucleus
g21423	258	27,408	9.55	Nucleus
g23438	467	50,378	5.23	Nucleus
g25319	308	32,858	8.50	Nucleus
g25339	303	33,578	8.45	Nucleus
g25726	253	25,782	9.13	Nucleus
g28923	269	29,061	5.05	Nucleus
g32088	447	48,074	8.89	Nucleus
g32327	291	31,451	9.76	Nucleus

## Data Availability

As of writing this manuscript, both genome and raw transcript data used in this study are under process of submission to NCBI. The genomic contigs are under submission “SUB10728242” and the raw transcript data under bioproject “PRJNA784246”.

## Supplementary Materials

The following supporting information can be downloaded at: https://www.mdpi.com/article/10.3390/genes13122235/s1, Figure S1: *E. uniflora DOF* genes genetic structure.

## Abbreviations

The following abbreviations are used in this manuscript:

AA	Amino-acid
AF	Atlantic Forest
CDS	Coding sequence
DEG	Differentialy expressed genes
*DOF*	DNA-binding with one-finger
CDF	Cycling-dof-factor
FC	Fold-change
PP	Posterior probability
RE	Restinga
RF	Riparian Forest
TF	Transcription Factor
MCOG	Majorly Conserved Orthologous Group
